# Genetics of biologically based psychological differences

**DOI:** 10.1098/rstb.2017.0162

**Published:** 2018-02-26

**Authors:** Hannah Sallis, George Davey Smith, Marcus R. Munafò

**Affiliations:** 1 MRC Integrative Epidemiology Unit, University of Bristol, Bristol, UK; 2 UK Centre for Tobacco and Alcohol Studies, School of Experimental Psychology, University of Bristol, Bristol, UK; 3 Centre for Academic Mental Health, Population Health Sciences, Bristol Medical School, University of Bristol, Bristol, UK

**Keywords:** heritability, GWAS, behavioural traits, personality, temperament, Mendelian randomization

## Abstract

In recent years, substantial effort has gone into disentangling the genetic contribution to individual differences in behaviour (such as personality and temperament traits). Heritability estimates from twin and family studies, and more recently using whole genome approaches, suggest a substantial genetic component to these traits. However, efforts to identify the genes that influence these traits have had relatively little success. Here, we review current work investigating the heritability of individual differences in behavioural traits and provide an overview of the results from genome-wide association analyses of these traits to date. In addition, we discuss the implications of these findings for the potential applications of Mendelian randomization.

This article is part of the theme issue ‘Diverse perspectives on diversity: multi-disciplinary approaches to taxonomies of individual differences'.

## 1. Introduction

Despite the early promise of behavioural genetic research, efforts to disentangle the genetic contribution to individual differences in behaviour (e.g. personality and temperament traits) have been slow. Early studies relied on a candidate gene approach to identify genes influencing these traits; however, many of these failed to replicate, despite having a plausible biological mechanism. More recent studies have used whole genome approaches to investigate the genetic architecture of behavioural traits. However, unlike for many other complex traits such as height [1,2] and schizophrenia [3], relatively few genetic variants have been identified that are robustly associated with temperament and individual differences in personality.

It has been argued that a small number of factors can be used to account for individual differences in personality [4–8]. Although there is no universally accepted framework of personality, the proposed factors (such as those suggested in Eysenck's five-factor model (FFM)) provide a good starting point when investigating the genetic architecture of these individual differences [7]. It is likely that individual molecular genetic effects associated with these traits will be small and large sample sizes will therefore be required to detect any association. By measuring specific traits, studies are able to harmonize phenotypes and pool analyses across cohorts. This will increase the power to detect genetic effects relevant to individual differences.

With recent advances in computing and availability of increasingly rich data sources, there has been a surge in the number of behavioural genetic studies investigating personality and temperament traits. Behavioural genetics has the potential both to quantify genetic influences on these traits and to indirectly quantify environmental influences [9]. Here, we provide a synthesis of these behavioural genetic studies to date. We begin by reviewing current work investigating the heritability of individual differences in personality and temperament traits; for the purposes of this review, we will refer to individual differences in these traits as simply ‘individual differences'. We also look at the results from genome-wide association analyses of these traits to date and discuss the implications of these findings for the potential for applying Mendelian randomization (MR) to individual differences in behaviour [10,11].

## 2. Estimating heritability

To investigate the genetic contribution to a phenotype, it is useful to first estimate its heritability (box 1). Heritability is the proportion of variation in a phenotype that can be attributed to genetic differences; these estimates are specific to the particular context and the timepoint at which they are estimated [13,15–17]. For example, if a trait has a heritability of 30%, then 30% of the variation in this trait is assumed to be due to genetic variation. However, although these estimates provide an idea of the size of the genetic component for a particular trait, they do not give us any information about which genes are likely to be responsible for it [18].

**Box 1. Heritability.**

Heritability is the proportion of variation in a phenotype (*V*_P_) that can be attributed to genetic differences for the particular context and timepoint at which it is estimated. This can include the proportion of variance due to additive genetic effects (*V*_A_), known as narrow-sense heritability (*h*^2^), or the proportion due to all genetic effects (*V*_G_), known as broad-sense heritability (*H*^2^) [12]. In addition to additive genetic effects, *H*^2^ includes both between-loci interactions (epistasis) and within-loci interactions (dominance) effects.
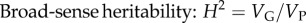
and
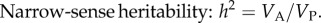

Heritability can be estimated using a number of methods. These include twin, family and adoption studies, in addition to more recent methods that use data captured by genome-wide arrays. Estimates calculated according to these models can be thought of as ‘true’ heritability. These estimates will incorporate heritability due to variants across the entire genome, including rare variants and those not captured by SNPs included on genotyping platforms. However, twin models, by design, use closely related individuals. These individuals are thus likely to share a great deal of their environment, which could lead to false inflation of heritability estimates.

Additional methods estimate heritability using SNP-based approaches. This includes heritability due to variants robustly associated with the phenotype of interest that are identified through genome-wide association study (GWASs) (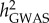), and heritability attributed to all SNPs captured on a GWAS array (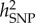). 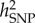 is typically larger than 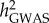, although 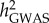 will increase with sample size as more variants are identified through GWASs, and is expected to approach 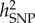 [13,14]. These approaches generally estimate heritability using unrelated individuals, which should minimize any false inflation due to shared environment, but do not give an estimate of true narrow-sense heritability because they do not include all additive genetic variance.

While the classic twin design can be used to estimate the proportion of phenotypic variation that can be attributed to genetic differences within a population, SNP-based methods can provide an estimate of the amount of variation that could potentially be explained using information from genome-wide SNP chips. These chips tend to encompass common variation and work by genotyping a fraction of the genome, in the hope that these SNPs will ‘tag’ the majority of the remaining SNPs. This 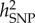 is an estimate of how much of the phenotypic variation could potentially be explained using the variants contained on these chips, rather than an estimate of ‘true’ *h*^2^. Any discrepancies between 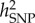 and the narrow-sense heritability estimated from twin studies may be attributed to variation explained by rare variants, small effect sizes not detectable using current sample sizes or common variation not tagged by SNPs included on the current chip.

### (a) Using twin studies to estimate heritability

Twin studies have been extensively used to disentangle the role of genetics and environment on human traits [19]. These models assume there are three distinct influences on phenotypic variation (*V*_P_) and these comprise additive genetic effects (*V*_A_), shared environmental effects (*V*_C_) and non-shared environmental effects (*V*_E_) [20]. Heritability estimates resulting from twin models are valid under certain assumptions. These assumptions include: (i) the twins are representative of the general population in terms of the trait, (ii) environmental effects are shared to the same extent by identical (MZ) and non-identical (DZ) twins, (iii) gene–environment interactions for the trait are minimal, and (iv) there is no assortative mating in the population [20]. Violations of these assumptions can result in biased (i.e. either increased or decreased) heritability estimates, and it seems reasonable that these assumptions could impact differently depending on the trait of interest. It should also be recognized that while one of the variance components is referred to as ‘non-shared environment’, this does not necessarily relate to environmental factors as they are usually understood, as the effects of somatic mutations, stochastic epigenetic changes and other random processes, as well as measurement error, are subsumed under this heading [13].

A recent meta-analysis by Polderman *et al.* [19] focusing on twin studies of human behavioural traits found that of all the phenotypes studied (more than 500 distinct traits), temperament and personality traits were among the top 10 most investigated. This study investigated the relative contribution of genetics and environment to a comprehensive list of traits studied over the previous 50 years, as well as assessing the presence of non-additive genetic effects. The authors suggest that of all the studies (*N* = 568) investigating temperament and personality traits, the majority (84%) of published twin correlations were consistent with a simple model containing just additive genetic effects [16,19]. However, many of studies in the meta-analysis did not report non-additive variance components, because these are generally (and arguably erroneously) constrained to zero when there is no ‘significant’ effect. This outcome is linked to sample size; thus, it is possible that this lack of non-additive effect is a result of the sample sizes analysed [16,21].

A recent meta-analysis of personality and temperament traits has combined evidence from twin, family and adoption studies [22]. The author, Vukasović & Bratko, find evidence for a heritable component to individual differences in personality, with heritability estimated at approximately 40%. The authors investigated the contribution of the different study designs and found that heritability estimates were consistently higher among twin studies than family and adoption studies. The authors also looked at potential moderating effects of gender and personality model on heritability. Although there is some suggestion in the literature that heritability differs according to gender [23–26], there was no strong evidence of a moderating effect when combining across all studies. Twin studies and Eysenck's theory of personality were both over-represented in the data; however, the authors only found evidence for a moderating effect of study design.

Despite the variation across models of personality structure considered in these studies, there is clear evidence that a proportion of variation in these individual difference traits can be attributed to genetic variation. Vukasović & Bratko [22] found no difference in heritability regardless of the model of personality used across studies. This is somewhat reassuring given the different models considered across cohorts and the ongoing development of personality theory.

**Figure 1.**
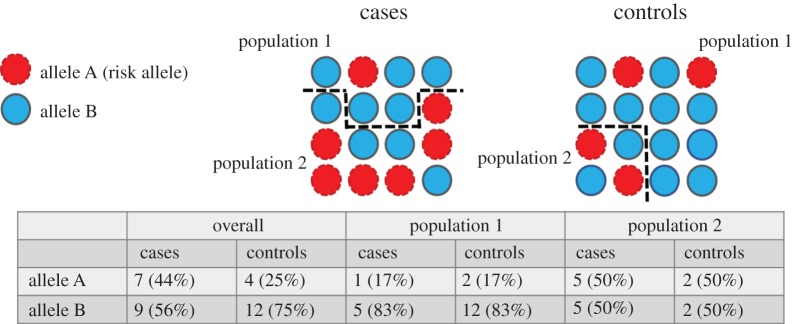
Population stratification. When looking at the sample overall, there is a higher frequency of risk allele A in cases than controls. However, there are two ‘hidden populations’ within this sample and the risk allele has a higher frequency in one population than the other. Cases and controls are sampled disproportionately, resulting in a false positive association for this variant. (Online version in colour.)

An additional consideration when undertaking genetic analysis is the population under investigation. In this meta-analysis, there is an underrepresentation of Asian populations and no representation of South American or African populations. Performing genetic analyses across different samples can be difficult owing to the possibility of population stratification (box 2 and figure 1). Risk alleles can occur with different frequencies across populations, and this can induce spurious associations. The population from which a sample has been drawn, therefore, needs to be taken into consideration to ensure that any observed association is not simply due to differences in allele frequency (see *ALDH2* Mendelian randomization example in section 5).

**Box 2. Population stratification.**

Allele frequencies can differ across populations, which can cause difficulties in association studies (figure 1). These differences in ancestry, or underlying population substructure, need to be taken into account to prevent finding associations that are due to this population substructure rather than to phenotype-associated variants. Methods of accounting for this include restricting samples to populations of common ancestry, adjusting results for a genomic inflation factor (*λ*) [27] or performing principal component analysis to account for variation in the data due to population differences, and adjusting the regression model for these components [28].

### (b) Single-nucleotide polymorphism-based approaches to estimating heritability

Although the twin studies discussed previously provide estimates of true narrow-sense heritability (*h*^2^), it can be problematic to obtain large enough samples to precisely estimate these effects. With recent advances in computing, and the increasing willingness of many cohorts to combine efforts through large-scale consortia, it has been possible to create increasingly large genotyped samples with data on a wide range of phenotypic measures. Unlike twin studies which rely on assumed levels of genetic correlation between twin pairs, we are now able to measure the degree of genetic similarity between individuals in a dataset and look at the extent to which genetic and phenotypic similarity correlates. This means that it is now possible to calculate heritability estimates using non-related individuals [14,29–33]. Methods have also been developed that use summary statistics from genome-wide association studies (GWASs) to calculate heritability estimates even when individual-level data are not available. These techniques use information on the correlation or linkage disequilibrium (LD) structure across the genome to estimate the heritability of a trait from GWAS summary statistics [34]. Although these approaches incorporate the LD structure among SNPs, estimates could be biased if the distribution of causal variants is non-random with respect to LD. A potential solution is to stratify SNPs jointly by minor allele frequency and LD. This approach appears to provide unbiased estimates of 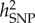 [14,33].

A recent study by Power & Pluess [35] used the FFM of personality to assess heritability of individual differences in behaviour in a sample of 5011 participants from the 1958 National Child Development Study (NCDS). They applied genome-wide approaches to estimate the heritability of each factor included in this model and found negligible estimates, with the exception of neuroticism (*h*^2^ = 0.15, s.e. = 0.08) and openness (*h*^2^ = 0.21, s.e. = 0.08). They also identified substantial genetic correlation (*r*_G_) between the neuroticism and extraversion factors (*r*_G_ = 0.82, s.e. = 0.39) and between neuroticism and openness (*r*_G_ = 1.00, s.e. = 0.50).

Although previous studies have estimated SNP heritability estimates for various facets of personality [36,37], this was the first study that attempted to estimate heritability for each of the FFM domains simultaneously. The estimates from this study are much smaller than the heritability estimated by twin studies (approximately 40%), leading the authors to suggest that common variants account for only around a quarter of causal genetic variation [35].

A recent meta-analysis by Lo *et al.* [38] estimated heritability based on GWAS summary statistics and found estimates that were consistent for both neuroticism and openness, and an increased heritability estimate for extraversion. Power to detect smaller *h*^2^ estimates was increased in this study, which involved a sample size of approximately 59 000 participants.

Table 1 shows the 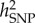 from neuroticism with increasing sample size. Heritability remains approximately 10%, with increasing precision as the sample size improves.

**Table 1.** Neuroticism heritability estimates from recent GWASs.

study	sample	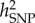	*N*
Bae *et al*. [39]	LLFS	0.252, *p* = 1.7×10^−15^	4595
de Moor *et al*. [40]	NTR	0.147, *p* = 0.02	3599
	QIMR	0.157, *p* = 0.18	3369
Smith *et al*. [41]	UK Biobank	0.136, s.e. = 0.015	91 370
Okbay *et al*. [42]	UK Biobank	0.091, s.e. = 0.007	170 910
Lo *et al*. [38]	23andMe	0.119, s.e. = 0.016	59 206
Luciano *et al*. [43]	UK Biobank	0.108, s.e. = 0.005	329 821
Nagel *et al*. [44]	UK Biobank	0.100, s.e. = 0.003	449 484

## 3. Identifying genetic associations

Despite emerging evidence that individual difference traits are heritable, so far relatively few individual genetic variants associated with these traits have been identified. This is likely due to the polygenic nature of these traits. Under the evolutionary neutral model, most variants with large effects will be rare; the majority of phenotypic variation is likely to be due to common variants of small effect [45]. Initial studies investigating the genetic basis to individual difference traits relied on investigating candidate genes. With advancements in computing power, it has become possible to increase the scope of analyses to take into account the entire genome rather than focusing on a single candidate locus. While candidate gene studies were purely hypothesis-driven and required knowledge of the underlying biology, GWASs are instead hypothesis-generating and test for associations across the whole genome (box 3). Associations found here can inform future studies and provide valuable information on underlying mechanisms.

**Box 3. Genome-wide association studies.**

GWASs use a hypothesis-free method of identifying specific common variants associated with a phenotype. Analyses are performed for each SNP in the dataset, which can be either genotyped or imputed. This assumes that any causal SNPs will be either captured within the analysis or tagged by those that are included. GWAS performs a regression for each variant, with the appropriate method determined by the format of the phenotype of interest. The regression model includes the genotype at that SNP plus any other relevant confounding variables, such as principal components, in order to adjust for population stratification, or other covariates that could account for variation in the phenotype not acting through the SNP of interest.

To account for the extensive multiple testing performed by running these analyses across the genome, a Bonferroni corrected *p*-value has been calculated which accounts for the likely number of functional variants being tested. This is the widely used *p* < 0.05 level of ‘statistical significance’ divided by 1 million, an approximation of the number of independent tests carried out across the genome. The accepted level of genome-wide significance is therefore *p* < 5 × 10^−8^ [46]. This is then typically followed by replication in an independent, comparable sample.

### (a) Candidate gene studies

In a candidate gene study, the association between allele frequency at a particular variant and phenotype of an individual is investigated [47]. These variants are selected *a priori* based on some underlying knowledge of a plausible biological mechanism associated with a particular locus. In recent years, the associations between the serotoninergic and dopaminergic neurotransmitter systems have gained attention [48–50]; however, the evidence for these associations has been somewhat inconsistent.

Although candidate gene studies were once the norm for behavioural genetic studies, many findings failed to replicate leaving the literature awash with false positive results. A recent study investigated findings from the largest schizophrenia GWAS to date and found no evidence that variants in the most-studied candidate genes were more associated with the disorder than non-candidate genes [51]. Candidate gene studies suffered from issues such as small sample sizes and lack of power, confounding due to population stratification which was frequently unaccounted for, and selective reporting of ‘positive’ results [52]. As a result, there has been a move away from these analyses in favour of the more agnostic GWAS approach.

### (b) Genome-wide association studies

#### (i) Analyses using the five-factor model of personality

GWASs have been successful in identifying loci associated with many complex traits, for example a recent GWAS of schizophrenia identified 108 loci robustly associated with the disorder [3], while over 200 susceptibility loci have been identified for inflammatory bowel disease [53]. Progress with regard to personality and temperament traits, however, has been slower.

A number of early GWASs focused on the FFM [39,54–57]; however, these were generally small and the majority of reported associations failed to replicate. Recently, a number of larger studies have used data from the UK Biobank to increase sample sizes [38,41–44]. The largest study to date, which focuses on five broad personality domains, is a recent meta-analysis of several GWASs which included data from 23andMe, Genetics of Personality Consortium, deCODE and UK Biobank (*N* = 123 132 to 260 861) [38]. This study identified six genetic loci, five of which were novel.

The majority of genome-wide analyses of individual differences have been performed in samples of European ancestry. However, studies have also investigated the genetic architecture of these factors within a Korean sample [54,55]. These studies also focused on an FFM of individual differences, but used a version of the NEO-PI revised for use in the Korean population. With the exception of rs2146180, an intergenic variant on chromosome 9, which associated with the openness domain among a sample of young Korean females, there were no robustly associated genetic variants identified among these samples.

#### (ii) Analyses of specific traits

In addition to studies looking at each of these five domains, other studies have focused on a particular trait. Several studies have focused on neuroticism, with the largest study to date including data from UK Biobank, 23andMe and the Genetics of Personality Consortium (*N* = 449 484). This study reports finding 136 independent genetic variants associated with neuroticism levels [44]. This suggests that for complex traits, for which there are likely to be many genetic effects of small size, increases in sample size are likely key to identifying genetic effects.

Other studies have focused on extraversion, although the samples for these have been smaller and to date have yielded few robust findings [58,59]. However, as shown in the heritability literature, there appears to be substantial genetic correlation between the traits of neuroticism and extraversion [35]. Extraversion also appears to be one of the more heritable traits, so it is possible that these analyses are currently underpowered. As of yet, there are no phenotypic measures available in the larger cohorts (such as UK Biobank) that would allow a GWAS of similar magnitude to be carried out.

Excitement-seeking and temperament were also investigated using genome-wide association analyses; however, these also comprised relatively small samples and did not find robust evidence of variants associated with these traits [60–62].

With the availability of large samples such as UK Biobank, there have been recent GWAS successes for other individual difference traits such as depressive symptoms. Progress in identifying genetic variants associated with depression had previously been slow, but in recent years, three large GWASs of major depressive disorder have been published [42,63,64]. The latest of these reports 44 independent risk loci for depression [64]. Reported heritability estimates for depression are similar to those reported for the FFM. It seems feasible that with similar increases in sample size, analyses of behavioural traits will be powered to detect genetic variants of small effect, and we may yet see comparable success in detecting variants that influence individual differences in personality and temperament.

## 4. Interpreting genome-wide association study findings

With recent advances in computing and the large number of cohorts willing to pool resources, there has been a rapid growth in the number of published GWASs and publicly available summary statistics. These studies have led to the identification of a vast number of genetic variants associated with disease outcomes in addition to health and lifestyle behaviours. Genome-wide association analyses have made a vast contribution to our understanding of the biological mechanisms unpinning many complex traits [1–3,65–67]. However, although GWAS findings often represent direct genetic effects, the possibility that they reflect the effects of modifiable risk factors should not be overlooked [68]. An illustrative example is the nicotinic receptor gene cluster *CHRNA5-A3-B4* on chromosome 15, which is robustly associated with heaviness of smoking. This association was first reported in candidate gene studies [69] and later identified through a GWAS of smoking quantity [70] and studies of nicotine metabolite levels [71–73]. Subsequent functional work suggests that this gene cluster plays a pivotal role in nicotine dependence by reducing the aversive effects of nicotine [74]. While this variant explains some of the biological underpinnings of this trait, the *CHRNA5-A3-B4* locus has also been identified in GWASs of lung cancer [75] and chronic obstructive pulmonary disease [76]. In the case of these traits, it is likely that the observed association with the *CHRNA5-A3-B4* locus is moderated through smoking status and mediated through the resulting tobacco exposure among smokers [68,71].

For individual personality and temperament traits, it may also be possible to identify the effects of modifiable risk factors by dissecting the results of newly published genome-wide analyses. It is likely that any effects, acting either through biological mechanisms or via modifiable exposures, are small, and large sample sizes will be required to detect these effects. With the recent release of the full UK Biobank sample, in addition to the increasingly collaborative direction in which genetic research appears to be heading, it seems likely that future analyses of these individual difference traits will be undertaken on greater samples, thus increasing power. Despite the fact that samples may not currently be powered to detect variants associated with modifiable risk factors at a genome-wide level, it is possible to interrogate the summary statistics relating to these analyses and investigate for enrichment of variants and networks that are known to relate to exposures of interest.

GWASs of other individual difference traits such as reproductive outcomes (measured by age at first birth and number of children ever born) [77] and educational attainment [78] have been successful in providing insights into the genetic architecture of these other behavioural phenotypes. Meta-analyses of GWASs of both age at first birth and number ever born have been conducted, and several independent loci have been identified that are robustly associated with either one or both of these traits [77]. The related genes have been identified as those that play a role in human reproduction and infertility. In addition, a large GWAS of educational attainment has identified 74 variants associated with years of schooling. These variants are disproportionately found in regions that regulate gene expression in the fetal brain [78]. These studies illustrate that well-powered studies can identify potentially relevant biological pathways, even for proximal phenotypes or behavioural phenotypes that at first appear to be largely environmentally driven.

### (a) Genetic overlap: what does this tell us?

Once we have estimated the heritability of a trait, we know that there is a genetic component to this phenotype, although further work needs to be carried out to determine which genes influence this. If heritability has been calculated for multiple traits, then we know that there is a genetic component to each of these; however, we do not know if these are influenced by the same genes. Genetic correlation is a method to quantify how much genetic overlap there is between traits. It provides an estimate of the additive genetic effect shared between pairs of traits. It is possible to estimate genetic correlation using either publicly available summary statistics or individual-level data. The vast number of published GWASs means that effect sizes are available for a wide range of traits on specific variants, and we can use the correlation between these to assess the genetic overlap between traits. Although these do not provide information on the causality of any potential relationship, they shed light on the amount of shared genetic architecture across traits. Any overlap here could be due to pleiotropy (genetic effects on multiple traits), shared biological mechanisms between traits or a causal relationship from one trait to another, but the direction of this cannot be ascertained from these approaches.

There is a well-documented association between personality domains and a range of health behaviours and physical and mental health outcomes [67,79–85]. Recent studies have investigated the extent to which there is shared genetic architecture between personality and temperament traits and both mental and physical health [43,44,83,85]. Nagel *et al.* investigated genetic overlap between neuroticism and several mental health outcomes, anthropometric and health-related traits using previously published summary statistics. Strong evidence of genetic correlation was found for several of these outcomes, with the greatest correlations observed with anxiety, depression and subjective well-being. There was also evidence of moderate–low genetic correlation between neuroticism and a number of other outcomes including schizophrenia, attention deficit hyperactivity disorder (ADHD), anorexia nervosa, educational attainment and height [44].

## 5. Mendelian randomization

**Figure 2.**
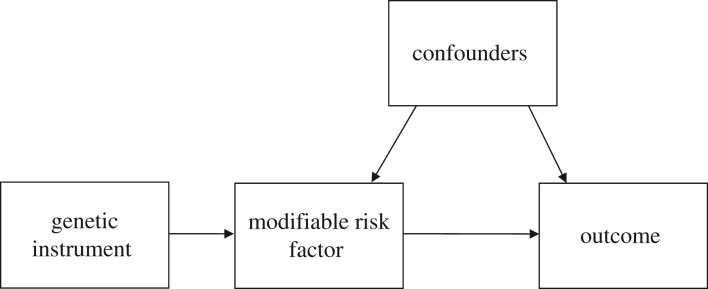
Directed acyclic graph illustrating Mendelian randomization.

Once genetic correlation between traits has been established, it may be possible to disentangle the nature of the relationship using techniques such as Mendelian randomization (MR). MR is a method of investigating causality using observational data. Genetic variants are used as proxies, or instrumental variables, for a modifiable risk factor of interest. The principles and assumptions underlying MR have been described in detail elsewhere [10,11,86,87]. Assuming these assumptions hold, there should be no association with potential confounders and analyses should not be subject to reverse causation (figure 2). MR can therefore be used to investigate the causality and direction of observational associations where there is a strong genetic instrument for the modifiable risk factor.

For cases where there are strong genetic instruments for both the exposure and outcome, bidirectional MR methods can be used to provide stronger evidence of the direction of effect. Assuming that the instruments are equally as strong for the exposure and outcome, if we were to observe strong evidence of an association in one direction, but not in the other, this would strengthen our confidence that this is the true direction of effect. However, MR alone is not enough to provide definitive evidence of causality, and evidence from different approaches should be triangulated. MR estimates should be compared with those from other methods to investigate whether results are consistent across different approaches and to build a more complete picture of the true effect.

To date, there have been relatively few MR studies carried out using individual differences in personality, in part due to the lack of strong instruments for these traits. However, with the publication of increasingly large GWASs for these traits, we expect instrument strength to increase and MR analyses to become more feasible. MR has provided insights into the causal effects of other individual difference traits, such as educational attainment, depression and anxiety [88,89]. A recent MR suggests that additional education is protective against the risk of coronary heart disease, with similar causal associations with reduced smoking, body mass index and improved blood lipid profiles [88]. MR analyses investigating the effect of smoking behaviours on depression and anxiety have found no strong evidence of an effect in this direction [89].

As mentioned previously, allele frequencies can differ across populations, and this information can be useful when designing MR analyses. A recent study investigating the role of alcohol use in adolescent internalizing and externalizing problems was performed within a sample of Chinese adolescents [90]. The ALDH2*2 genotype is found almost exclusively in Asian populations and can be used as a strong instrument for alcohol use in these samples. This study found evidence of a causal role for alcohol in adolescent aggression and attention problems.

In addition, MR can provide insights into evolutionary developmental hypotheses as it allows us to look at varying levels of genetic vulnerability to a trait, rather than focusing on an individual's phenotype. It seems likely that particular traits would be susceptible to selection; for example, individuals with a diagnosis of schizophrenia tend to have lower reproductive success [91,92]. However, the disorder remains at a constant prevalence in the population. This is likely due to favourable characteristics that are linked to some genetic vulnerability for the disorder that, although elevated, is not high enough for the phenotype to manifest.

## 6. Further research directions

An alternative to increasing sample size is to refine the phenotype being investigated. Reducing the noise in a phenotype measure may lead to a clearer genetic signal, and thus, lower sample numbers would be required to detect this. Attempts to refine the phenotype are useful in terms of improving statistical power, but could limit the utility of any findings if results are not generalizable to the whole population. Therefore, there is a trade-off between statistical power and clinical utility. Ideally, studies combining the large sample sizes of resources, such as UK Biobank, with the detailed phenotyping of smaller cohort studies would provide the greatest power to detect genetic effects, if such effects exist. Longitudinal cohorts have the potential to contribute to this; measurement error will be reduced for longitudinally defined phenotypes, resulting in increased power.

Although there is ongoing discussion about the best model of individual differences in personality, the factor structure suggested by Eysenck and others is a useful starting point when investigating the genetic architecture of these traits [4–7]. Work has been done to investigate the impact of using different personality models when attempting to unpick the genetic structure of these traits, and little evidence of a difference across models has been found [22]. There is some suggestion that a general factor of personality should be constructed [93,94]. Although there is little work that has been done to specifically investigate the genetic architecture of such a factor [95], the genetic correlation between various factors in the FFM provides some evidence for this theory [35]. Further work should be done to investigate the influence of using different models of these individual differences.

To date, the majority of studies investigating underlying genetics of behavioural traits have focused on samples of European ancestry. Individual molecular genetic effects are likely to be of small size, and large samples will be required to identify them. There is a finite number of samples available with relevant phenotypes and genetic data available; therefore, in order to maximize power and sample size, combining samples across populations is likely to play an important role in future genetic studies. Analytical approaches that can account for underlying population stratification will be required.

## 7. Conclusion

Despite ongoing debate about the structure of individual differences in personality, there is clear evidence of a genetic component to these traits. Consistent heritability estimates have been calculated for a number of domains using both twin studies and whole genome approaches. Increasingly large GWASs have been published which identify a number of genetic variants for individual differences in behavioural traits. To date, the largest study of a personality trait focuses on neuroticism, which has identified 136 variants of small effect. As the number of samples with both genetic and phenotypic data increases, it is likely that more such evidence will emerge. This has the potential both to provide insights into potential biological mechanisms and to generate instruments for use in future MR analyses investigating causal consequences of these individual differences in personality.

## Data accessibility

This article has no additional data.
