# Correction to ‘Genetics of biologically based psychological differences’

**DOI:** 10.1098/rstb.2018.0173

**Published:** 2018-04-23

**Authors:** Hannah Sallis, George Davey Smith, Marcus R. Munafò

*Phil. Trans. R. Soc. B* **373**, 20170162. (Published Online 26 February 2018) (doi:10.1098/rstb.2017.0162)

## 

It has been brought to our attention that our article ‘Genetics of biologically based psychological differences’ contained some minor errors and ambiguities that we would like to correct. A list of corrections are as follows.

In the Introduction (paragraph 2), sentence 2 should read: *‘*Although there is no universally accepted framework of personality, the proposed factors (such as those suggested by Eysenck and in the five-factor model (FFM)) provide a good starting point when investigating the genetic architecture of these individual differences [4–7].’

In Box 1 (paragraph 2), sentence 3 should read: ‘Estimates calculated according to these family-based models can be thought of as ‘true’ heritability.’

In the same paragraph, sentence 6 should read: ‘These individuals are thus assumed to share a great deal of their environment, but if the equal environments assumption is violated this can impact on heritability estimates; for example, more similar treatment of identical (MZ) twins could lead to false inflation of heritability estimates.’

In Box 1 (paragraph 3), sentence 1 should read: ‘Genome-wide methods estimate heritability using SNP-based approaches.’

In the same paragraph, sentence 4 should read: ‘These approaches generally estimate heritability using unrelated individuals, which should minimize confounding due to shared environment, and follow the same experimental design as GWASs [26], but do not give an estimate of true narrow sense heritability because they do not include all additive genetic variance.’

In Box 1 (paragraph 4), sentence 4 should read: ‘Any discrepancies between 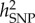 and the narrow sense heritability estimated from twin studies may be attributed to variation explained by rare variants or common variation not tagged by SNPs included on the current chip.’

In section 2a (paragraph 1), sentence 6, we should refer to reference [16] not [13].

In the same section, paragraph 3, sentence 2 should read ‘The authors, Vukasović & Bratko, find evidence for a heritable component to individual differences in personality, with heritability estimated at approximately 40%.’

In section 2b (paragraph 1), sentence 6 should read: ‘These techniques use information on the correlation or linkage disequilibrium (LD) structure across the genome to estimate the SNP heritability 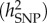 of a trait from GWAS summary statistics (see Box 1 for a definition of 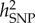) [34].’

In the same section, paragraph 1, sentence 9 should read ‘This approach appears to provide unbiased estimates of 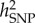 [14].’

In table 1, the study by Power & Pleuss should be included:

**Table:** 

Power & Pleuss [35]	NCDS	0.150, s.e. = 0.08	4924

In section 5 (paragraph 5), sentence 4 should read ‘This is likely due to favourable characteristics that are linked to some genetic vulnerability for the disorder in those who do not succumb to the outcome.’

In section 6 (paragraph 2), sentence 1 should read: ‘Although there is ongoing discussion about the best model of individual differences in personality, the factor structures suggested are a useful starting point when investigating the genetic architecture of these traits [4–7].’

While these amendments do not change the conclusions of our article, we are grateful for the opportunity to correct them.

